# Association between age at first childbirth and type 2 diabetes in Chinese women

**DOI:** 10.1111/jdi.13073

**Published:** 2019-06-12

**Authors:** Xueqi Qu, Hanyu Wang, Shiyuan Zhou, Zhe Fang, Jingyuan Li, Kun Tang

**Affiliations:** ^1^ Bloomberg School of Public Health Johns Hopkins University Baltimore Maryland USA; ^2^ Research Center for Public Health Tsinghua University Beijing China; ^3^ Institute for Medical Humanities Peking University Health Science Center Beijing China; ^4^ School of Public Health Peking University Health Science Center Beijing China

**Keywords:** Childbirth, China, Type 2 diabetes

## Abstract

**Aims/Introduction:**

The present study aims to explore the relationship between age at first childbirth and developing type 2 diabetes, and analyze potential modifiers of its relationship.

**Materials and Methods:**

The data were obtained from the baseline survey of a large‐scale cohort study in 10 diverse areas of China. Multivariate logistic regressions were used to analyze the association between developing type 2 diabetes and the age at first childbirth. Adjustments were made in each model on sociodemographic, socioeconomic and reproductive covariates. Subgroup analyses were further carried out to investigate the effects of reproductive factors, body mass index at 25 years of age and hypertension on the above‐mentioned association.

**Results:**

Women who have their first child at age <20 years (odds ratio [OR] 1.33, 95% confidence interval [CI] 1.22–1.44) and between the ages of 20 and 24 years (OR 1.16, 95% CI 1.10–1.23) were more likely to be diagnosed with type 2 diabetes compared with those who have their first child between the ages of 25 and 29 years; equal to or more than three parities (OR 1.44, 95% CI 1.30–1.60), premenopausal status (OR 1.58, 95% CI 0.99–2.54), overweight at age 25 years (OR 1.62, 95% CI 1.37–1.93) and hypertension (OR 1.37, 95% CI 1.22–1.53) are all conditions found to increase the odds of developing type 2 diabetes in those who have their their first child at an early age (<20 years).

**Conclusions:**

The study concluded that having the first child at an early age is associated with an increased likelihood of developing type 2 diabetes later in life; reproductive factors, overweight at age 25 years and status of hypotension could modify such an association.

## Graphical abstract

Women who delivered their first child at <20 years‐of‐age were more strongly associated with developing type 2 diabetes later in life, compared with those who had their first child between 25 and 29 years‐of‐age. After adjustments were made for socioeconomic characteristics, lifestyle, reproductive factors and physiological risk factors, there remained a significant inverse association between age at first birth and developing type 2 diabetes. In addition, reproductive factors (i.e., parity, menopause status), hypertension and body mass index at age 25 years were found to have an effect on such an association.

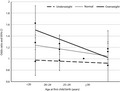

## Introduction

In the past decades, there has been a trend of delayed age at first childbirth in China, especially in urban areas^1^. Both socio‐environmental and biological factors might have an influence on the timing of childbearing age. For example, women with high education qualifications tend to delay transitioning into motherhood^2^. The age at menarche, which marks sexual maturity and the beginning of the reproductive span, biologically determines the appropriate age at childbirth. Childbearing induces a series of physiological and socio‐environmental transitions in a women's life that could have a long‐term impact on a woman's health. Therefore, as important as the time point of childbearing is, age at first childbirth might also be an important predictor of health and well‐being for women.

There is an increase in the body of literature that focuses on the association between age at first childbirth and its long‐term health outcomes for mothers. Yet, whether an early age at first childbirth is a protective factor or a risk factor for chronic diseases still remains uncertain. Early motherhood is associated with poor self‐rated health and higher rates of mortality hazarda^3^, ^4^. In addition, women who bear their first child at an earlier age are more likely to have cardiovascular diseases^5^ and higher blood pressure^6^. Nevertheless, with regard to cancer, later age at first childbirth is related with an increased risk of pancreatic cancer^7^ and breast cancer^8^.

Young age at first childbirth (<19 years or 18 years) has also been found to be associated with a higher risk of diabetes^9^, ^10^, and diabetes‐related mortality is higher among teenage mothers^11^. However, previous studies were carried out in limited populations, and the external validity of these findings in other populations were still uncertain. Although one international study collected data from Canada, Albania, Colombia and Brazil, showing consistent results, the sample size of that particular study was relatively small (*n* = 1,040) and only included women aged between 65 and 74 years^10^. For Chinese populations, there is a natural gap in literature to understand the characteristics of age at first childbirth and its long‐term health consequences. Therefore, the present study aimed to analyze the association between the age at first childbirth and developing type 2 diabetes in Chinese women, and to explore the role of sociodemographic, socioeconomic, lifestyle and reproductive factors that might potentially modify such an association.

## Methods

### Participants

The present study used the baseline data from a population‐based cohort study among 0.5 million people carried out from 2004 and 2008 in 10 diverse areas of China. A detailed design of this study has been described elsewhere^12^. Briefly, 512,891 participants aged 30–79 years were recruited from five urban and five rural regions in China. The selection of the regions in this survey was based on the local patterns of disease and exposure to specific risk factors, population stability, quality of death, and disease registries. The questionnaire of this survey consisted of 10 sections, and investigated participants’ demographic characteristics, socioeconomic status, personal and family medical history, reproductive history, and lifestyle information (such as alcohol consumption and diet). After completing the questionnaire by a laptop‐based data‐collecting system, qualified physicians carried out physical examinations and collected a blood sample in order to obtain more health information. Furthermore, a quality control survey was carried out after a few weeks of the baseline survey to monitor the quality of the collected data from several aspects, such as recruitment rate and so on. Before entering the survey, informed consent was obtained from all participants. According to the aim of the present study, only female respondents with at least one live birth were included in the analysis.

The present study was approved by the institutional review boards at Oxford University and the China National Center for Disease Control. All the participants included in this study provided informed consent, and all study procedures were carried out in accordance with the Declaration of Helsinki ethical principles for medical research.

### Measures

In the questionnaire, female participants were asked about the number of live births, and then for each live birth, their age at the end of the pregnancy. Twins were considered as one childbirth. The respondents were coded into four categories according to the age at the end of the pregnancy of the first live birth: <20 years, 20–24 years, 25–29 years and ≥30 years. Diabetes status was dichotomized into “yes” or “no” based on whether they self‐reported as having been clinically diagnosed with diabetes before. We excluded those who were diagnosed before the age of 18 years from analyses to avoid misclassification of type 1 diabetes. Participants who were diagnosed before, at the same year of or 1 year after first childbirth were also excluded from the analysis for the consideration of temporality and influence from gestational diabetes mellitus. Among those who were not previously diagnosed with type 2 diabetes, a random plasma test was carried out using Johnson & Johnson Sure Step Plus Meter (LifeScan, Milpitas, CA, USA). Participants with a glucose level ≥140 mg/dL and <200 mg/dL were invited to return the next day for fasting plasma glucose testing. Participants with a random plasma glucose ≥200 mg/dL or fasting plasma glucose ≥126 mg/dL were identified as screen‐detected type 2 diabetes patients.

Socioeconomic, lifestyle and reproductive covariates were assessed, including age, region, education, household income, current occupation, alcohol consumption, tobacco use, metabolic equivalents of task (MET), parity, age at menarche, menopause status, body mass index (BMI) at aged 25 years and systolic blood pressure (SBP). Level of education was categorized into primary school and below, middle/high school, and college and above. The household income was coded into three levels: <10,000 Yuan (1 USD = 6.3 Yuan), 10,000–34,999 Yuan and ≥35,000 Yuan. Agriculturally‐related and factory workers were classified as manual labor, whereas the other types of occupation were grouped as non‐manual labor (i.e., administrator/manager, professional/technical, sales/service workers, retired, housewife/househusband, self‐employed, unemployed and others that were not stated). According to participants’ self‐reported alcohol consumption and tobacco use frequency, both were grouped into “never regular” or “ex/currently regular.” The metabolic equivalent hours per day, which reflects the amount of daily physical activities, was assessed according to the types and duration of physical activities associated with work, commuting, household chores and leisure‐time exercises during the past 12 months. The amount of daily physical activity (in metabolic equivalent hours per day [MET‐h/day]) was then classified into three groups: <13 h/day, 13–26 h/day and >26 h/day. As for the parity, participants were classified as “had one live birth,” “had two live births” and “had three or more live births.” Menopausal status was dichotomized into “yes” or “no.” As many studies believe that the normal age of menarche ranged from 13 to 18 years, we categorized participants into three groups based on age at menarche: <13 years, 13–18 years and >18 years. Family history of diabetes, which was a dichotomous variable, was accessed by asking whether a family member (fathers, mothers and siblings) had diabetes. BMI at age 25 years was calculated to reflect the maternal physical situation close to the time of the first childbirth. BMI at age 25 years was calculated through the measurement of the standing height by physical examination as well as the self‐reported weight at age 25 years. According to standards, BMI at age 25 years was classified as underweight (<18.5 kg/m^2^), normal (18.5–25 kg/m^2^) and overweight (≥25 kg/m^2^). Finally, the SBP was measured during physical examination, and participants were dichotomized into two subgroups according to whether the mean SBP was <140 mmHg or equal to or above.

### Statistical analysis

Descriptive statistics were used to report the baseline characteristics of the participants’ age at first childbirth, while at the same time including the socioeconomic characteristics, lifestyle and reproductive factors, BMI at age 25 years, and SBP in the analysis. The associations between the different ages at first childbirth and developing type 2 diabetes were estimated as odds ratios (ORs) and 95% confidence intervals (95% CI) by logistic regression. Crude regression models and other adjusted models were applied step‐by‐step. Six logistic regression models were fitted with: (i) unadjusted; (ii) adjusted for age, region, income, education and occupation; (iii) added adjustment for alcohol consumption, tobacco use and MET; (iv) added adjustment for reproductive factors, including parity, age at menarche and menopausal status; (v) added adjustment for BMI at age 25 years and mean SBP; and (vi) added adjustment for family history of diabetes. Furthermore, subgroup analysis was carried out, and ORs were analyzed in each subgroup of parity, menopausal status, age at menarche, BMI at age 25 years and SBP. The control group for all analyses were among those who had their first childbirth aged 25 to 29 years. The adjusted means of age difference (in years) between diabetes diagnosis age and the age of first live birth was presented in Appendix 1. The association between parity and developing type 2 diabetes was analyzed (Appendix 2). All statistical analyses were carried out using Stata 15 for Windows (StataCorp, College Station, TX, USA) with statistical significance level set at *P *< 0.05.

## Results

Overall, 297,641 women had at least one live birth in the present study, and their mean age was 51 years. The mean age at first birth was 23 years, and more than half of the women had their first child aged between 20 and 24 years. The overall prevalence of being diagnosed as type 2 diabetes was 5.37%. Sociodemographic factors, socioeconomic characteristics, lifestyle and reproductive history distribution of the study population across different groups of age at first birth are shown in Table 1. The mean age for women who had their first child aged <20 years was highest across four groups. Most of the women (83.28%) who had their first childbirth at or >30 years came from urban areas, whereas 20.64% of women with first childbirth aged <20 years were from urban areas. The proportion of participants with a college education was the lowest in the group of having first childbirth aged <20 years compared with other groups. A total of 19.27% of women who had their first child between 25 and 29 years were from a family with an annual household income of >35,000 Yuan. The highest proportion of working in manual labor appeared in the group aged <20 years. As for lifestyle, 77.55% of women with first childbirth aged <20 years did not drink alcohol regularly, while the distributions of smoking behaviors and MET level were similar across all groups of age at first childbirth. The highest proportion of participants having more than three parities (74.38%) and postmenopausal status (97.26%) were found in the group that had their first child aged <20 years. While participants with first childbirth aged ≥30 years had the highest proportion of having menarche aged <13 years (7.34%). The distribution of BMI among the three groups was similar, with a slight high prevalence of underweight among participants with first childbirth aged ≥30 years (5.07%). The proportion of participants with a family history of diabetes was 3.50%, 5.65%, 10.50% and 11.20%, among those with first childbirth aged <20 years, 20–24 years, 25–29 years and >30 years, respectively. The prevalence of hypertension was higher among those with first childbirth aged <20 years (45.12%). The prevalence of type 2 diabetes was 4.97% in the group of age at first childbirth between 20 and 24 years, and 7.89% in the group of age at first childbirth <20 years.

**Table 1:** Characteristics of participants by age at first childbirth

	<20 years	20–24 years	25–29 years	≥30 years
	*n* = 28,141	*n* = 171,330	*n* = 87,238	*n* = 10,932
Sociodemographic factors				
Mean age, years (SD)	58.80 (9.74)	50.18 (10.62)	50.11 (9.13)	51.01 (10.56)
Urban (%)	20.64	34.10	67.14	83.28
Socioeconomic factors				
Education (%)				
Primary school and below	91.57	63.34	37.25	25.11
Middle/high school	8.29	35.14	53.27	58.45
College and above	0.14	1.52	9.49	16.44
Income, Yuan (%)				
<10,000	40.49	32.69	21.36	21.40
10,000—34,999	45.73	52.03	59.37	58.47
≥35,000	13.78	15.28	19.27	20.13
Occupation (%)				
Manual labor	79.15	71.10	42.67	23.56
Lifestyle factors				
Alcohol assumption (%)				
Never/regular drinker	77.55	65.87	55.54	58.68
Tobacco use (%)				
Never regular smoker	91.31	94.94	96.03	96.55
MET, h/day (%)				
<13	41.32	33.46	38.01	46.14
13–26	31.36	34.28	37.29	36.06
≥26	27.31	32.27	24.71	17.80
Reproductive factors				
Parity (%)				
1	3.28	27.65	54.37	73.81
2	22.34	36.00	29.43	19.20
≥3	74.38	36.35	16.20	6.99
Postmenopausal (%)	97.26	91.86	87.85	91.41
Age at menarche, years (%)				
<13	4.45	5.00	6.30	7.34
≥13 to ≤18	94.47	88.84	87.29	86.75
>18	1.09	6.15	6.41	5.92
Ever taken oral contraceptives (%)	8.10	10.42	10.08	6.49
BMI (%)				
<18.5	5.97	4.23	3.59	5.07
≥18.5 to <25	58.99	61.98	61.00	61.67
≥25	35.04	33.80	35.41	33.26
With family type 2 diabetes history (%)	3.50	5.65	10.50	11.20
Hypertension (%)	45.12	26.80	22.18	20.93
Type 2 diabetes (%)	7.89	4.97	5.25	6.12

BMI, body mass index; MET, metabolic equivalents; SD, standard deviation.

The association between age at first childbirth and developing type 2 diabetes is shown in Table 2. In the fully adjusted model (model 6), women in the groups who had their first childbirth aged <20 years (OR 1.33, 95% CI 1.22–1.44) and between 20 and 24 years (OR 1.16, 95% CI 1.10–1.23) were more likely to be diagnosed with type 2 diabetes compared with those with a first childbirth aged between 25 and 29 years, whereas having the first childbirth ≥30 years was not found to be associated with developing type 2 diabetes (OR 1.10, 95% CI 1.00–1.23). Although the intensity of association changed with adjustments for the different factors, the association remained positive in all models. Women with an age at first childbirth <20 years had higher odds of developing type 2 diabetes in various models (model 1 OR 1.54, 95% CI 1.46–1.63; model 2 OR 1.32, 95% CI 1.24–1.41; model 3 OR 1.31, 95% CI 1.23–1.40; model 4 OR 1.33, 95% CI 1.25–1.43; model 5 OR 1.30, 95% CI 1.20–1.41; model 6 OR 1.33, 95% CI 1.22–1.44), after adjusting for all potential confounders.

**Table 2:** Association between age at first childbirth and developing type 2 diabetes

Age (years)	Model 1	Model 2	Model 3	Model 4	Model 5	Model 6
	OR (95% CI)	OR (95% CI)	OR (95% CI)	OR (95% CI)	OR (95% CI)	OR (95% CI)
<20	1.54 (1.46–1.63)	1.32 (1.24–1.41)	1.31 (1.23–1.40)	1.33 (1.25–1.43)	1.30 (1.20–1.41)	1.33 (1.22–1.44)
20–24	0.94 (0.91–0.98)	1.12 (1.08–1.17)	1.12 (1.07–1.16)	1.16 (1.10–1.21)	1.14 (1.08–1.20)	1.16 (1.10–1.23)
25–29	1	1	1	1	1	1
≥30	1.17 (1.08–1.28)	1.03 (0.94–1.12)	1.01 (0.93–1.10)	1.09 (1.00–1.20)	1.08 (0.98,1.20)	1.10 (1.00–1.23)

Model 1: unadjusted; model 2: adjusted for age, region, education, income and occupation; model 3: plus adjustment for tobacco use, alcohol assumption and metabolic equivalents; model 4: plus adjustment for reproductive history, including parity, menopausal status and age at menarche; model 5: plus adjustment for body mass index at age 25 years and mean systolic blood pressure; model 6: plus adjustment for family history of diabetes. CI, confidence interval; OR, odds ratio.

Table 3 shows the effects of different reproductive factors on the association between age at first birth and developing type 2 diabetes. It appeared that the number of births might strongly affect the association. For women who had only one child, the ORs for developing type 2 diabetes are not significantly different across various ages at first birth groups. However, for those with three or more children, the ORs for developing type 2 diabetes decreased from 1.44 (95% CI 1.30–1.60) among those who delivered their first child aged <20 years to 1.01 (95% CI 0.76–1.35) among those who delivered their first child aged ≥30 years. The association between age at first childbirth and developing type 2 diabetes was similar among the three age at menarche groups. Having the first child aged <20 years was associated with an increased likelihood of developing type 2 diabetes among both premenopausal (OR 1.58, 95% CI 0.99–2.54) and postmenopausal women (OR 1.33, 95% CI 1.22–1.45). However, the strength of association appeared stronger in the postmenopausal group.

**Table 3:** Relative odds of age at first childbirth with developing type 2 diabetes stratified by reproductive factors

	<20 years	20–24 years	25–29 years	≥30 years
Parity†				
1	0.92 (0.49,1.72)	1.14 (1.00–1.30)	1	1.19 (1.02–1.38)
2	1.22 (1.02–1.46)	1.18 (1.08–1.28)	1	0.91 (0.75–1.11)
≥3	1.44 (1.30–1.60)	1.16 (1.07–1.27)	1	1.01 (0.76–1.35)
Age at menarche^(years)^ ‡ ^,^ §				
<13	1.13 (0.96–1.34)	1.10 (0.98–1.22)	1	1.01 (0.83–1.23)
≥13 to ≤18	1.38 (1.21–1.58)	1.24 (1.14–1.36)	1	1.12 (0.94–1.34)
>18	1.28 (1.09–1.52)	1.16 (1.05–1.30)	1	1.20 (0.97–1.49)
Menopausal status§,¶				
Yes	1.33 (1.22–1.45)	1.17 (1.10–1.23)	1	1.07 (0.96–1.19)
No	1.58 (0.99–2.54)	1.20 (0.95–1.51)	1	1.75 (1.20–2.54)

^†^Adjusted for age, region, education, income, occupation, tobacco use, alcohol consumption, metabolic equivalents (MET), menopausal status, age at menarche, body mass index (BMI) at age 25 years, mean systolic blood pressure (SBP) and family history of type 2 diabetes. ^‡^Adjusted for age, region, education, income, occupation, tobacco use, alcohol consumption, MET, parity, menopausal status, BMI at age 25 years, mean SBP and family history of type 2 diabetes. ^§^Adjusted for age, region, education, income, occupation, tobacco use, alcohol consumption, MET, menopausal status, age at menarche, parity, BMI at age 25 years, mean SBP and family history of type 2 diabetes. ^¶^Adjusted for age, region, education, income, occupation, tobacco use, alcohol consumption, MET, age at menarche, parity, BMI at age 25 years, mean SBP and family history of type 2 diabetes.

**Figure 1:**
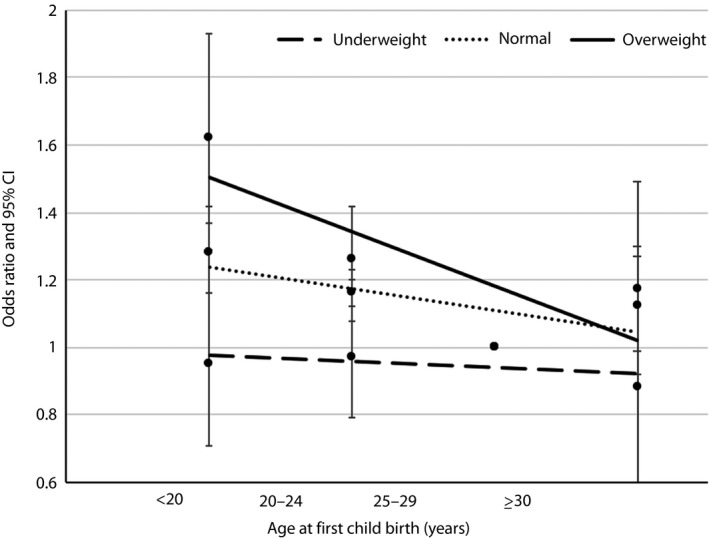
Odds ratio (95% confidence interval [CI]) for developing type 2 diabetes by age at first childbirth and by body mass index at age 25 years. Adjusted for age, region, education, income, occupation, tobacco use, alcohol consumption, metabolic equivalents of task, menopausal status, age at menarche, parity, mean systolic blood pressure and family history of diabetes

**Figure 2:**
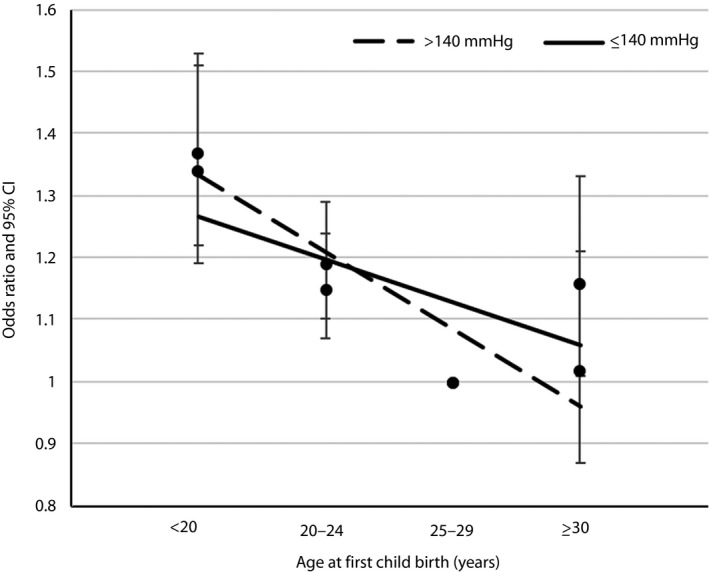
Odds ratio (95% confidence interval [CI]) for developing type 2 diabetes by age at first childbirth and by systolic blood pressure. Adjusted for age, region, education, income, occupation, tobacco use, alcohol consumption, metabolic equivalents of task, menopausal status, age at menarche, parity, body mass index at 25 years and family history of diabetes.

For BMI, the stronger association between age at first childbirth and developing type 2 diabetes was found among the overweight group, with ORs decreased from 1.62 (95% CI 1.37–1.93) to 1.17 (95% CI 0.92–1.49) across various age at first childbirth groups (Figure 1). The association between age at first childbirth and developing type 2 diabetes was stronger among participants who had hypertension, with ORs decreased from 1.37 (95% CI 1.22–1.53) to 1.02 (95% CI 0.87–1.21), as shown in Figure 2.

## Discussion

The present study suggests that women who delivered their first child aged <20 years were more strongly associated with developing type 2 diabetes later in life compared with those who had their first child aged 25–29 years. After adjustments were made for socioeconomic characteristics, lifestyle, reproductive factors and physiological risk factors, there remained a significant inverse association between age at first birth and type 2 diabetes. In addition, reproductive factors (i.e., parity, menopause status), hypertension and BMI at age 25 years were found to have an effect on such an association.

Before the present study, there was no study carried out in China to investigate the impact of age at first childbirth on the risk of developing type 2 diabetes. The results from the present study on the association between the age at first childbirth and type 2 diabetes filled the gap in the literature of such an essential issue in the Chinese population, which was useful for the screening and intervention of diabetes among Chinese women. The present study utilized the baseline data from a large‐scale population‐based prospective study with a total sample size of 0.3 million women. The high quality of data ensured a reliable analysis on the relationship between age at first childbirth and developing type 2 diabetes. It also provided an opportunity to explore the influence of other factors on the association. The present study is the first to discuss the influence of reproductive factors, BMI at age 25 years and SBP on the association between age at first childbirth and developing type 2 diabetes, which might provide unique angles and public health implications for such an association.

Admittedly, there are some limitations of the present study. First, the causal relationship between the age at first childbirth and type 2 diabetes was not sufficiently established because of the cross‐sectional nature of the present study, although respondents with diabetes diagnosed before first delivery were excluded from the present study. Thus, further prospective research is expected to be undertaken in order to assess the causality of such an association. Second, there was no distinction between type 1 diabetes, gestational diabetes mellitus and type 2 diabetes in the survey's questionnaire. Accordingly, bias might exist and influence the results of the study, the the three types of diabetes have different pathogenesis and risk factors. However, cases of diabetes diagnosed before 18 years‐of‐age, at the same year or 1 year after the time of first childbirth were excluded from the analysis in the study. This could minimize the influence of gestational diabetes mellitus and type 1 diabetes. Third, the present study was not designed as a nationally representative survey, therefore the result might not extrapolate to the entire Chinese population. Nevertheless, because of the large sample size the present study involved, it might still to a large extent reflect the situation in this particular population. Finally, BMI at age 25 years was used in the analysis instead of the total duration of obesity, which was not collected in the present study. Duration of obesity after delivery could potentially influence the future development of type 2 diabetes, which should be investigated in future studies.

The present results show that early age at first childbirth might be associated with type 2 diabetes. It is consistent with the findings from a Korean national study that reported that women aged <19 years at first childbirth were significantly at risk of developing diabetes, but not with impaired fasting glucose among postmenopausal women^9^. Although the mechanisms of the association remain uncertain, there are several possible pathophysiological pathways towards the relationship. First, pregnant teenagers are more likely to suffer from pre‐eclampsia compared with their older counterparts^13^. Women with a history of pre‐eclampsia could predict not only the incidence of hypertension during the postpartum period, but also the increased risk of developing diabetes after pregnancy, whether accompanied with gestational diabetes or not^14^.

Second, obesity, as one of the risk factors of type 2 diabetes, is used to explain the association between young age at first childbirth and developing type 2 diabetes in the previous study. A Korean study has shown that obesity in postmenopausal women is associated with first childbirth at a young age, which might be explained by gestational weight gain, change of fat storage mechanisms and lifestyle caused by childbearing^15^. However, in another Korean study, the association between age at first childbirth and developing diabetes was only found in the non‐obesity group instead of the obesity group^16^. In the present study, the association was found in women who were normal or overweight around the period of pregnancy, and the strength of the association appeared stronger in the overweight group compared with the normal group. However, the cross‐sectional design of the present study made it difficult to determine whether overweight women were more likely to have children at a younger age or if teenage mothers were more likely to become overweight after pregnancy. Therefore, future prospective studies should be carried out to explore the intermediating role of gestational weight gain and change of weight between pre‐pregnancy and post‐pregnancy periods among groups of different ages at first birth.

Third, certain metabolic mechanisms might provide explanations for the association between age at first birth and developing type 2 diabetes in later life. Several metabolic changes induced by pregnancy might have long‐term impacts on women, such as increased level of estrogen^17^ and triglyceride^18^. For example, the expansion of β‐cell mass occurs during pregnancy to maintain the normal blood sugar levels, which might induce insulin secretion dysfunction and diabetes later in life^19^. As for women who had a child at a young age, they might be exposed to such metabolic changes for a longer time compared with those who began parenthood at an advanced age. Therefore, the risk of developing type 2 diabetes increased for those young mothers. The findings of the present study also showed that women with multiparity might have a stronger association between the decrease in the age of first childbirth and the increased likelihood of developing type 2 diabetes. This piece of evidence somehow supports the hypothesis of metabolic effects, as multiparity extends the time of risky metabolic exposure brought about by pregnancy^19^.

Furthermore, adjustments made for socioeconomic status significantly attenuated the association between the age at first childbirth and type 2 diabetes, suggesting that those factors might have played an important role in the association. Evidence has shown that early motherhood is associated with low socioeconomic status. Women with lower socioeconomic status might lack the basic knowledge and access to family planning, and are more likely to experience unintended pregnancy or have children at an early age^20^, ^21^. In turn, early childbearing could predict women's subsequent poverty^22^. People with low socioeconomic status have been proved to be more likely to have type 2 diabetes^23^, ^24^, because of physical inactivity^25^, unhealthy food overconsumption^26^ and tobacco use^27^ among those people. Therefore, young mothers living in poverty might meet more challenges in their lives and have unhealthy lives, which might increase the risk of developing type 2 diabetes.

The present study found that having the first child at an early age is significantly associated with type 2 diabetes in women's adulthood. Furthermore, the association was stronger among women with lower socioeconomic status and multiparity. The findings suggest that teenage mothers, especially those who have their first childbirth aged <20 years, might be more vulnerable to type 2 diabetes later in their lives. Preventative measures and diabetes screening programs should be designed to target this particular population.

## Disclosure

The authors declare no conflict of interest.

## Adjusted means of age difference (in years) and 95% confidence interval between age at diabetes diagnosis (age at study date for those who were screen‐detected) and age at first live birth

**Table:**

Age at first childbirth (years)	<20	20–24	25–29	≥30
Adjusted means of age difference (95% CI)	30.22 (30.03–30.41)	37.43 (37.14–37.73)	34.16 (34.02–34.30)	25.23 (24.75–25.70)

Means are adjusted for age at study date, region, education, income, occupation, tobacco use, alcohol consumption, metabolic equivalents, menopausal status, age at menarche, parity, body mass index at age 25 years and family history of diabetes. CI, confidence interval.

## Association between parity and type 2 diabetes

**Table:**

Parity	1	2	3	≥4	Per additional live birth
Type 2 diabetes (95% CI)	1	1.29 (1.20–1.39)	1.35 (1.23–1.47)	1.26 (1.14–1.40)	1.06 (1.02–1.09)

Adjusted for age at study date, region, education, income, occupation, tobacco use, alcohol consumption, metabolic equivalents, menopausal status, age at menarche, age at first childbirth, body mass index at 25 years and family history of diabetes. CI, confidence interval.
